# Supplementary material for: Cell adhesion and fluid flow jointly initiate genotype spatial distribution in biofilms
Source: PLoS Comput Biol. 2018 Apr 16;14(4):e1006094. doi: 10.1371/journal.pcbi.1006094 (PMC5901778; doi:10.1371/journal.pcbi.1006094)
Supplement: S1 Text — Sensitivity analysis of the effect of system size in the simulations output. (DOCX) [file pcbi.1006094.s001.docx]

**Supplementary information for**

**Cell adhesion and fluid flow jointly initiate biofilm population structure**

R. Martínez-García, C. D. Nadell, R. Hartmann, K. Drescher, J. A. Bonachela

**S1 Text. Size effect analysis.**

Our experimental results have been obtained using a square observation window of lateral length *L* = 60*μm* embedded within a much larger microfluidic device. Using the simulation framework, we investigated whether the spatial measures in the occupation patterns are influenced by the size of the focal system.

First, we focused on the correlation length, for both highly-adhesive and weakly-adhesive strains, in the intense flow limit (*f* = 1). As shown in the main text, in this regime the model accurately reproduces experimental results if the same focal area is used in both approaches. Numerical simulations on larger systems confirm that both strains maintain the same qualitative trends across simulated areas, and although the curves are quantitatively affected by the simulated area, they intersect at the same value of the initial population density (Fig A1). The sublinear scaling of the correlation length with system size, suggests a saturation of the correlation length in the limit in which *ξ* << *L* for any initial density and cell adhesiveness (Fig A2). Next, we prepared a simulation setup in which we divided a system of lateral length *L =* 120*μm* in four tiles of lateral size 60*μm*, and simultaneously measured the correlation length in the total system and in each of the tiles. To ensure that the initial population density was constant for the whole system and each tile, we initialized every tile with a total population density *ρ­_0_*_­_ (*ρ­_0_*_­_/2 of each strain on average). Focusing on the intense flow limit (*f* = 1), the distance traveled by relocated cells in the direction of the flow is a random number between 0 and *L*, so for a given focal area, the population mixing depends on whether the system is isolated or embedded in a bigger one. However, the use of periodic boundary conditions, as discussed in the main text, minimizes differences in the correlation length for strong flows (Fig A3). The residual difference in the correlation length is due to the fact that, in small isolated systems, the periodic boundary conditions can introduce small additional correlations, since detached cells that exit the system through one of the borders and re-enter through the opposite may be relocated close to their original position. These events are equivalent to limited dispersal and hence tend to increase clonal cluster size. However, as it is shown in Fig A3, their effect is negligible, reinforcing the validity of our periodic boundary conditions.

Next, we extended our analysis to consider a *L*=240 *μm* patch with various flow intensities. In this scenario, system size influences the outcome of the simulations in two directions. First, the set of flow strengths for which patterns of weakly-adhesive cells have larger clonal clusters than those made by adhesive strains increases considerably. Second, such regions show a larger difference correlation length for bigger systems (Fig A4a). This result indicates that avoiding the production of adhesion substances does not entail a residual gain (slightly larger clusters without the metabolic cost of matrix production) but, for a wide range of environmental flows (*f* < 0.4), such gain can be very significant, as much as that of matrix-production in strong environmental flows (but, again, without the metabolic cost). If, on the other hand, we are observing a small system that, instead of in isolation, is within a bigger one, the flow range for which weakly-adhesive cells show larger clusters segregation is reduced to very weak intensities. This shrinkage of the region results from our flow strength implementation discussed above: when the small system is part of a bigger one, detached cells can travel larger distances even at weak environmental flows and thus the range of limited dispersal is reduced (Fig A4b compared to Figure 4). All this phenomenology indicates that, in a real system, the ratio between the typical distance travelled with the flow and the system size will influence considerably the quantitative (but not the qualitative) behavior of our measure for genetic segregation.

Finally, we analyzed the effect of the system size on cluster size variability for *σ* = 1 strains. The standard deviation of the correlation length maintained its concavity regardless of the system size, but it reached its maximum at different initial population densities (Fig A5a). Since highly-adhesive cells are not relocated by the flow, the confluence pattern is strongly determined by the spatial distribution of the founder population, and its correlation length variability depends on the variability of the initial lineage mixing. Hence, it is the number of cells and not the density what determines the position of the maximum in the standard deviation (Fig A5b). For high cell numbers, it is very unlikely to randomly create a configuration with large clusters, whereas for low cell numbers, the cluster size at confluence is necessarily large. In addition, for a fixed initial density (or number of cells) the standard deviation increases with system size since the variability in the spatial distribution of the founder population increases with system size.

In summary, the observation window can quantitatively affect some results of our analyses as well as the regions of the parameter space in which they are expected. Therefore, not only environmental forces, such as fluid flow, and bacterial traits, such as cell adhesion, are important to quantify biofilm population structure. The size of the observation frames needs to be accounted for as well. Importantly, however, the overall qualitative behavior of our results is not affected by the size of the observation window and, therefore, any conclusion drawn for smaller surfaces can be extrapolated to larger systems.

**
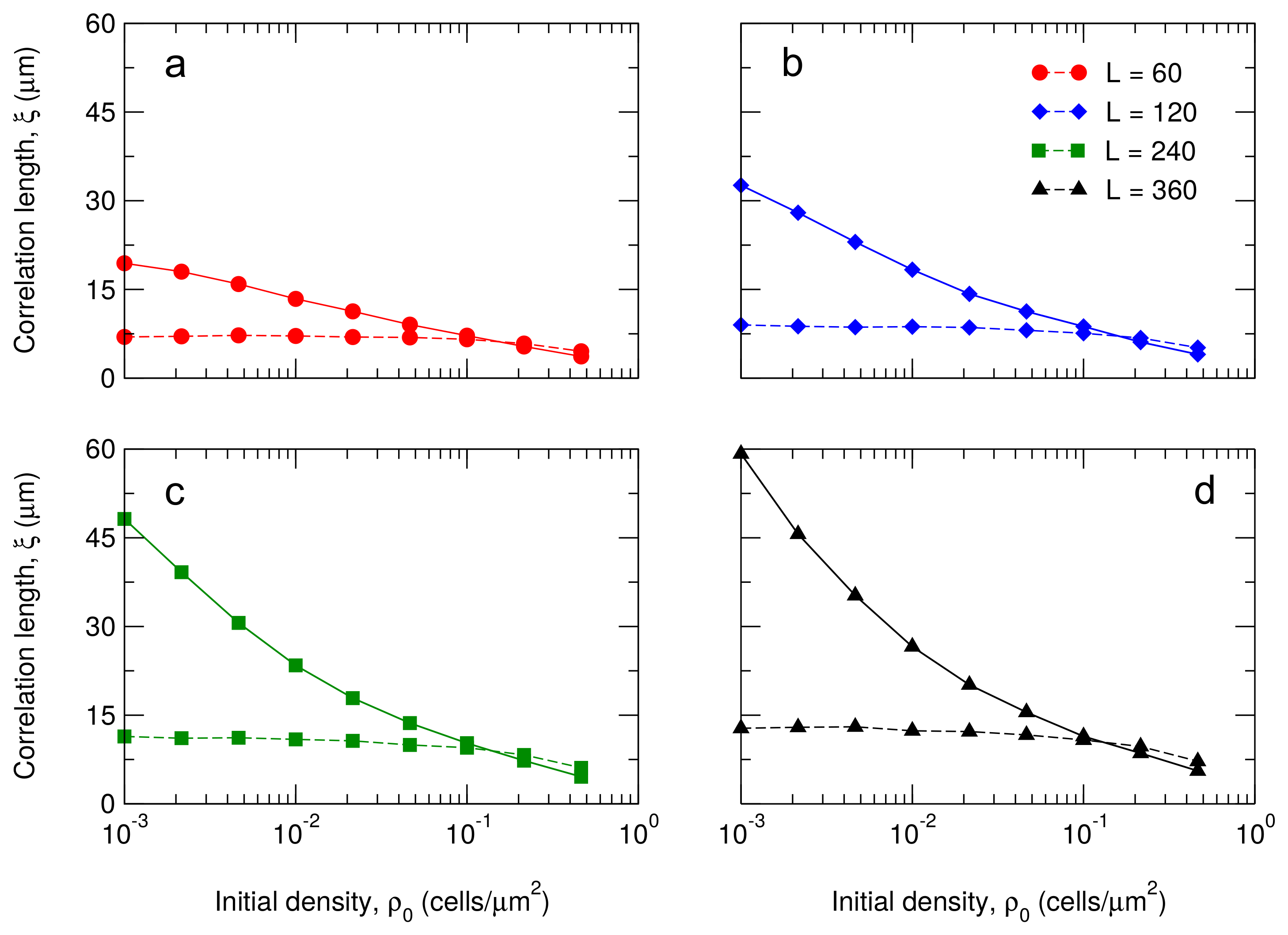
**

**Fig A1. Effect of surface in correlation length.** The clonal cluster size is strongly influenced by the extension of the colonized surface, although the trends of highly-adhesive and weakly-adhesive strains, and the crossing point between curves, are system size independent. Full lines correspond to *σ* = 1 and dashed lines to *σ* = 0. a) *L* = 60*μm*, b) *L* = 120*μm*, c) *L* = 240*μm*, d) *L* = 360*μm.*

**
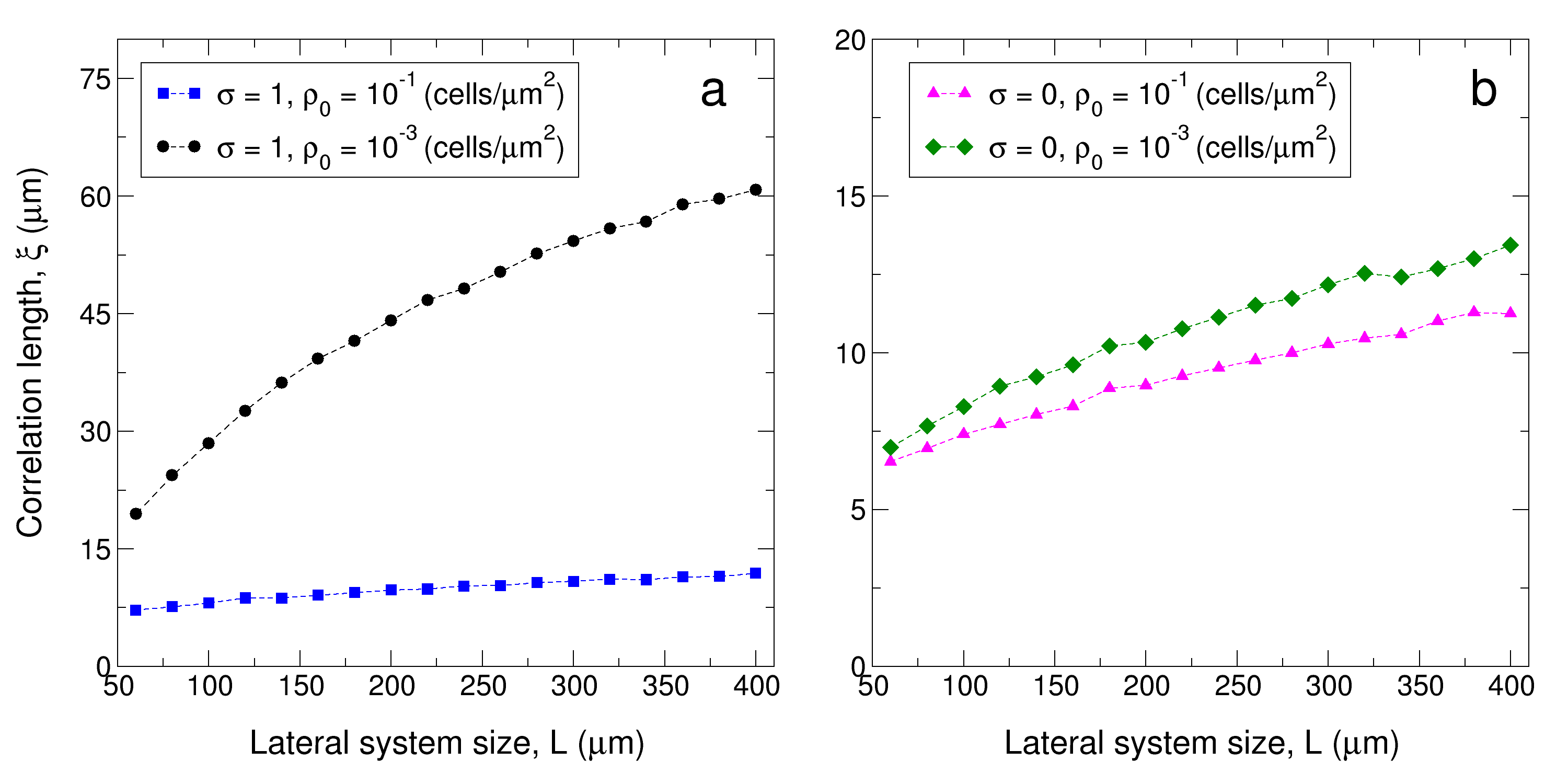
**

**Fig A2. Correlation length scaling with system size.** The correlation length scales sub-linearly, both for highly-adhesive (a) and weakly-adhesive (b) strains, with system size, which suggests a clonal cluster size saturation for large systems.

**
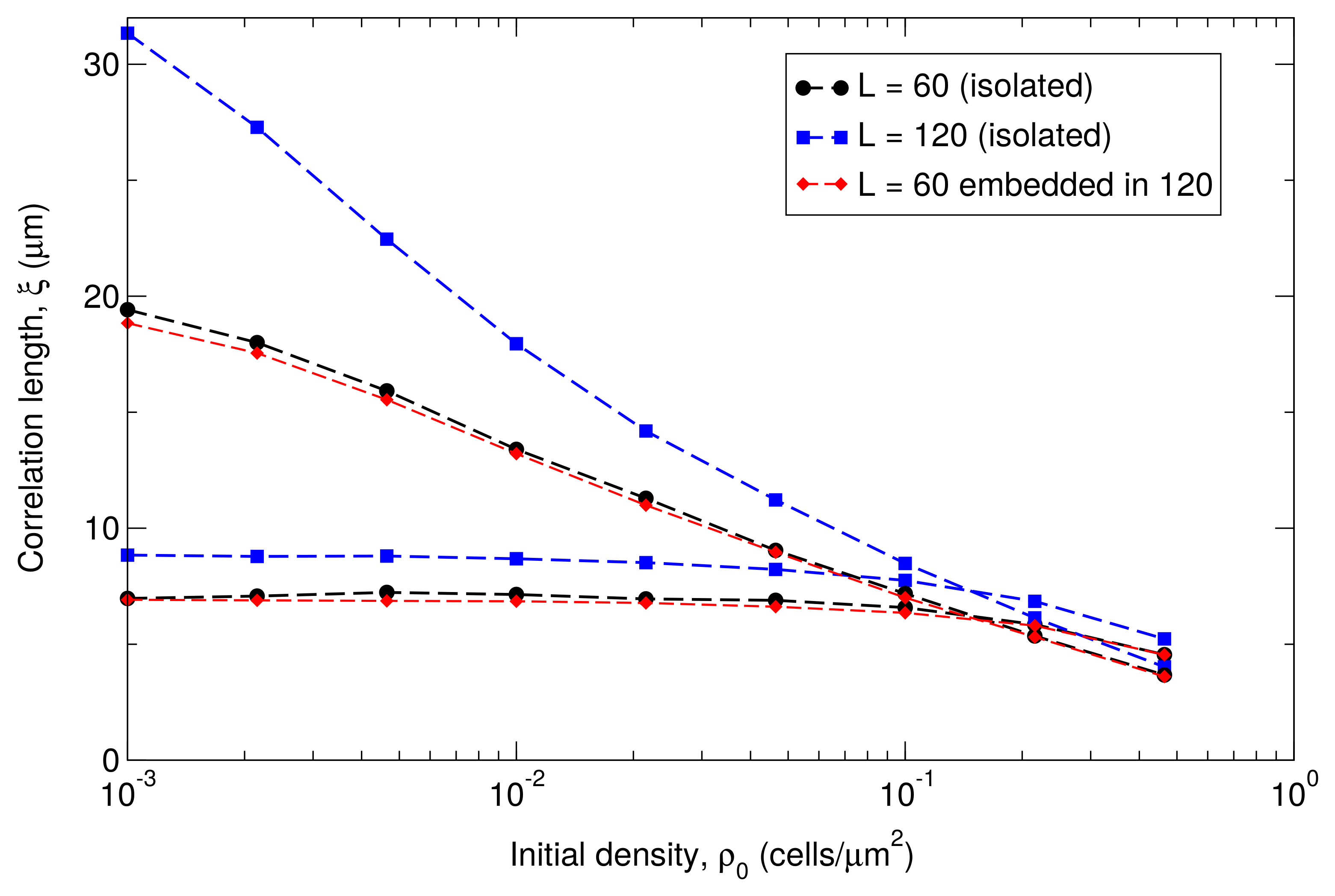
**

**Fig A3. Finite size effects in the correlation length.** Tiles within a larger system have the same correlation length than isolated surfaces of the same size. Simulations are run independently on systems of lateral length *L* = 60*μm* (black circles) and *L* = 120*μm* (blue squares). In this latter scenario, the system is divided in four tiles of lateral length 60*μm* and the correlation length of each of the tiles is independently obtained following the same protocol used in the *L* = 60*μm* case.

**
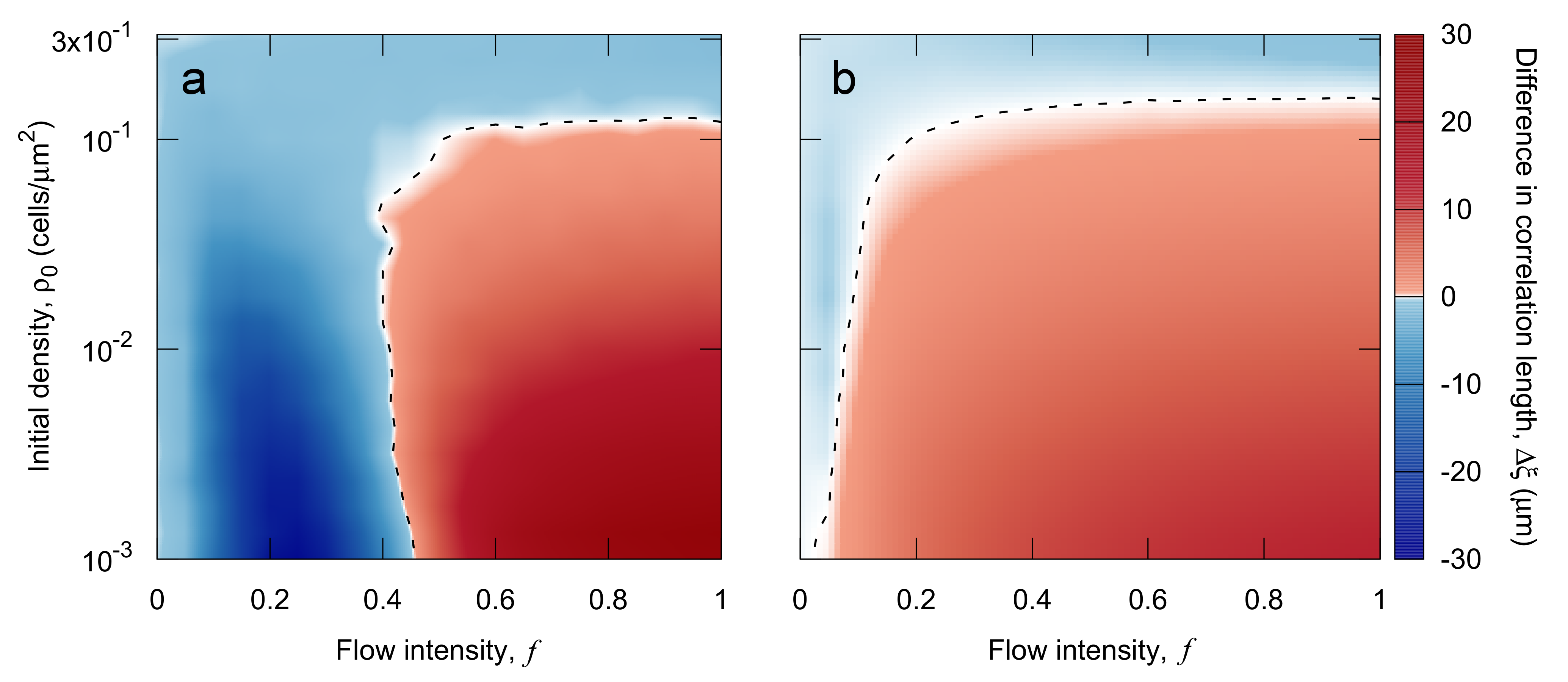
**

**Fig A4. System size effects on the correlation length difference between highly-adhesive and weakly-adhesive strains.** Correlation length differences are evaluated on a system of lateral length *L* = 240*μm* (b) and the result is compared to what would be observed using an observation window of lateral length *L* = 60*μm* within the system (a). In the latter case, each of the 16 observation windows is used as an independent replicate. Therefore, averages in b) are taken over 4000 replicates whereas 64000 independent realizations are gathered for the smaller system.

**
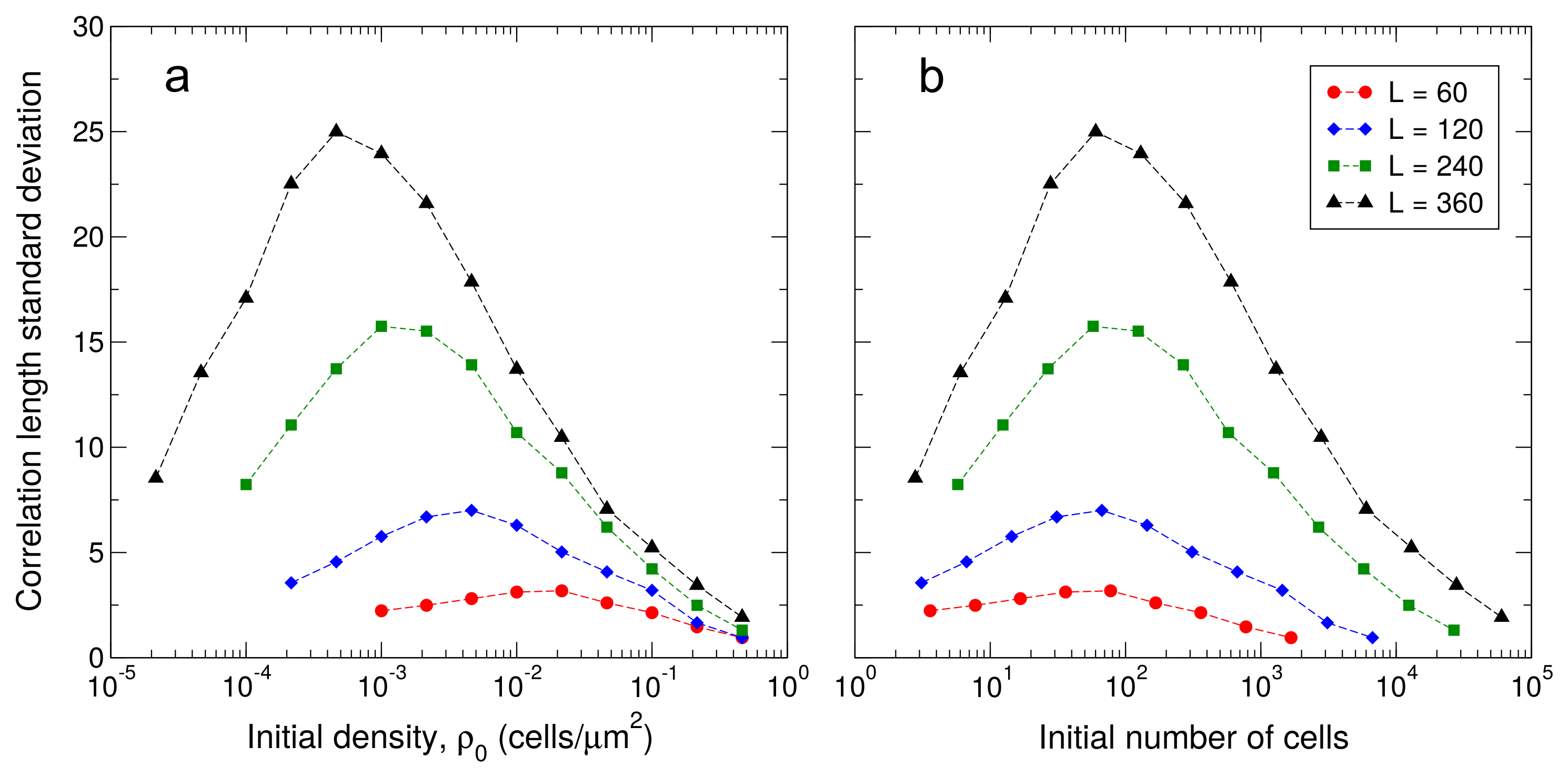
**

**Fig A5. Variability in the correlation length is influenced by system size.** Correlation length standard deviation versus initial population density (a), and initial number of cells (b). Color code is maintained in both panels
