# Supplementary material for: Cell adhesion and fluid flow jointly initiate genotype spatial distribution in biofilms
Source: PLoS Comput Biol. 2018 Apr 16;14(4):e1006094. doi: 10.1371/journal.pcbi.1006094 (PMC5901778; doi:10.1371/journal.pcbi.1006094)
Supplement: S1 Table — (DOCX) [file pcbi.1006094.s002.docx]

| **Symbol** | **Name** | **Cause** | **Value** |
| --- | --- | --- | --- |
| *σ* | Cell adhesiveness | BT | Free parameter in [0,1] |
| *f* | Flow intensity | EF | Free parameter in [0,1] |
| *ρ_0_* | Founder cell density | BT by EF interaction | Free parameter in [10^-3^, 0.5] *cells/μm^2^* |
| *μ* | Reproduction rate | BT | Fixed parameter, 0.57 a.u. |
| *L* | Lateral lattice size | EF | Fixed parameter, 60 *μm* |
| *dt* | Time step | -- | Fixed parameter, 1/L^2^ a.u. |
| *dx* | Lattice mesh | -- | Fixed parameter, 1 *μm* |
| *p_d_* | Detachment probability | BT by EF interaction | $f(1-\sigma)$ |
| *p_s_* | Shoving probability | BT by BT interaction | $\frac{1-\sigma}{2}$ |
| *Δx* | *x*-distance traveled | EF | Random, in [0, *fL*] |
| *Δy* | *y*-distance traveled | EF | Random, in [-*Δx*, *Δx*] |
