# Supplementary figures and images for: Cell adhesion and fluid flow jointly initiate genotype spatial distribution in biofilms
Source: PLoS Comput Biol. 2018 Apr 16;14(4):e1006094. doi: 10.1371/journal.pcbi.1006094 (PMC5901778; doi:10.1371/journal.pcbi.1006094)

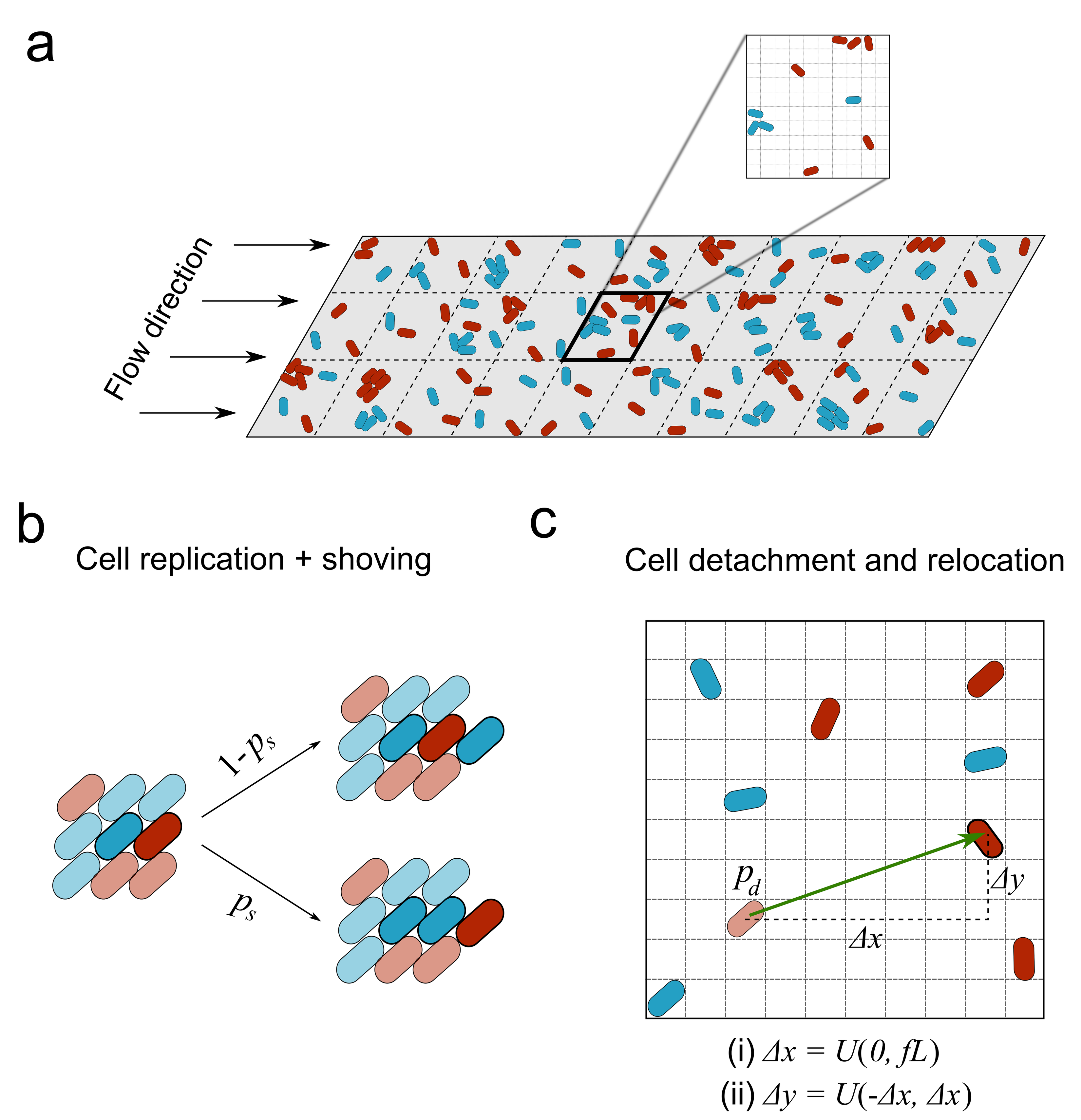

Supplement: S1 Fig — a) Experimental chamber, its tiles or viewing fields, and model representation of one of the tiles as a 2D lattice with one cell at each lattice box. b) Cell displacement due to shoving following cell division occurs with probability ps. With complementary probability 1-ps the resident cell keeps its position and the newborn jumps to one of the adjacent empty position. c) Cells may be detached from the surface of the chamber with probability pd and transported to a new emplacement following the relocation rules explained in the text with periodic boundary conditions (Materials and methods). U(a,b) indicates a uniformly distributed random variable between a and b. f is the flow intensity and L the lattice lateral length. (TIF) [file pcbi.1006094.s003.tif]

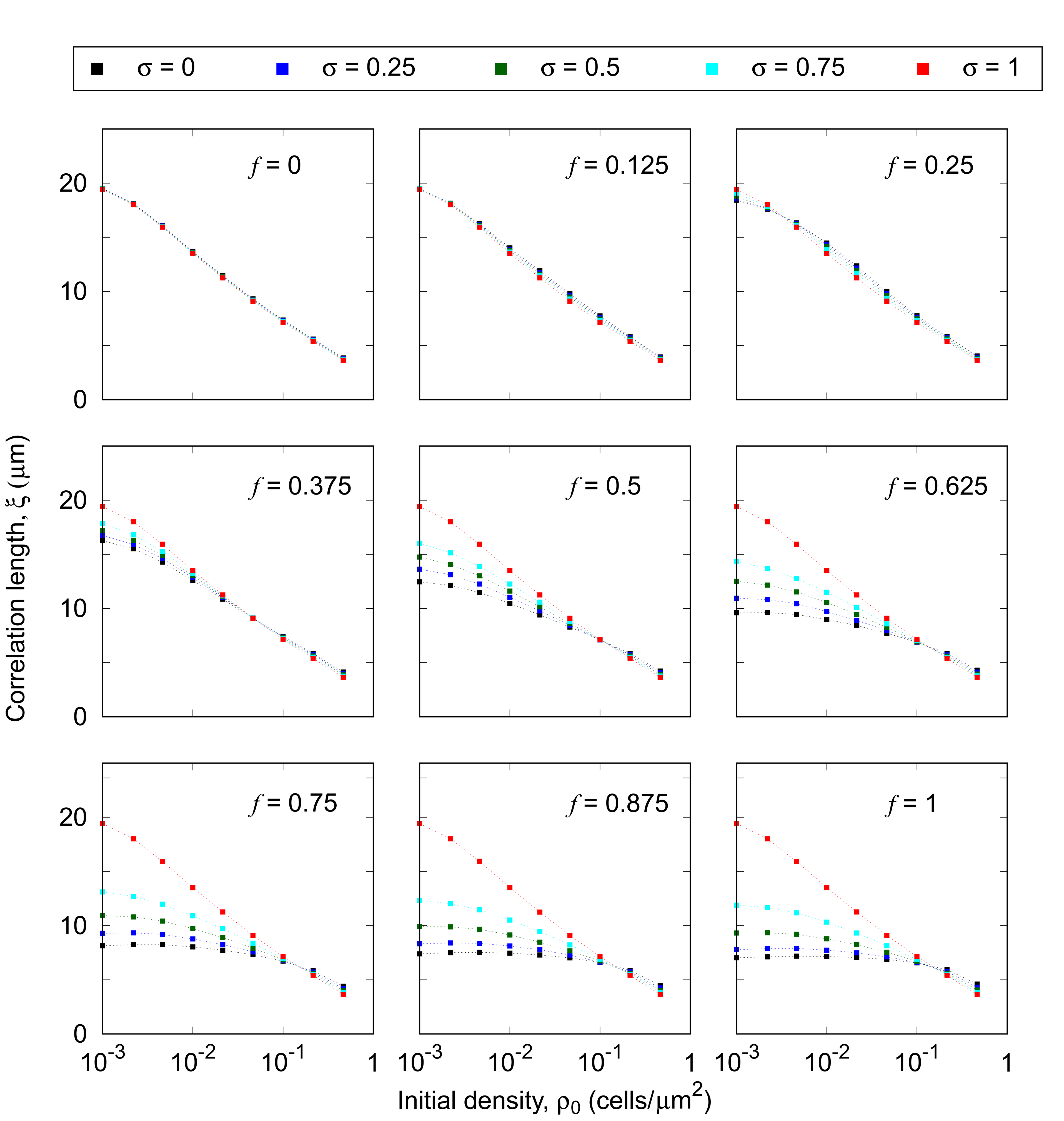

Supplement: S2 Fig — Mean correlation length, ξ, for different colonization strategies (σ, ρ0) in several ecological conditions given by the flow intensity f. Each curve represents a cell adhesiveness σ. The color code is maintained in all the panels. Averages are taken over 2x10^6^ independent model realizations. (TIF) [file pcbi.1006094.s004.tif]

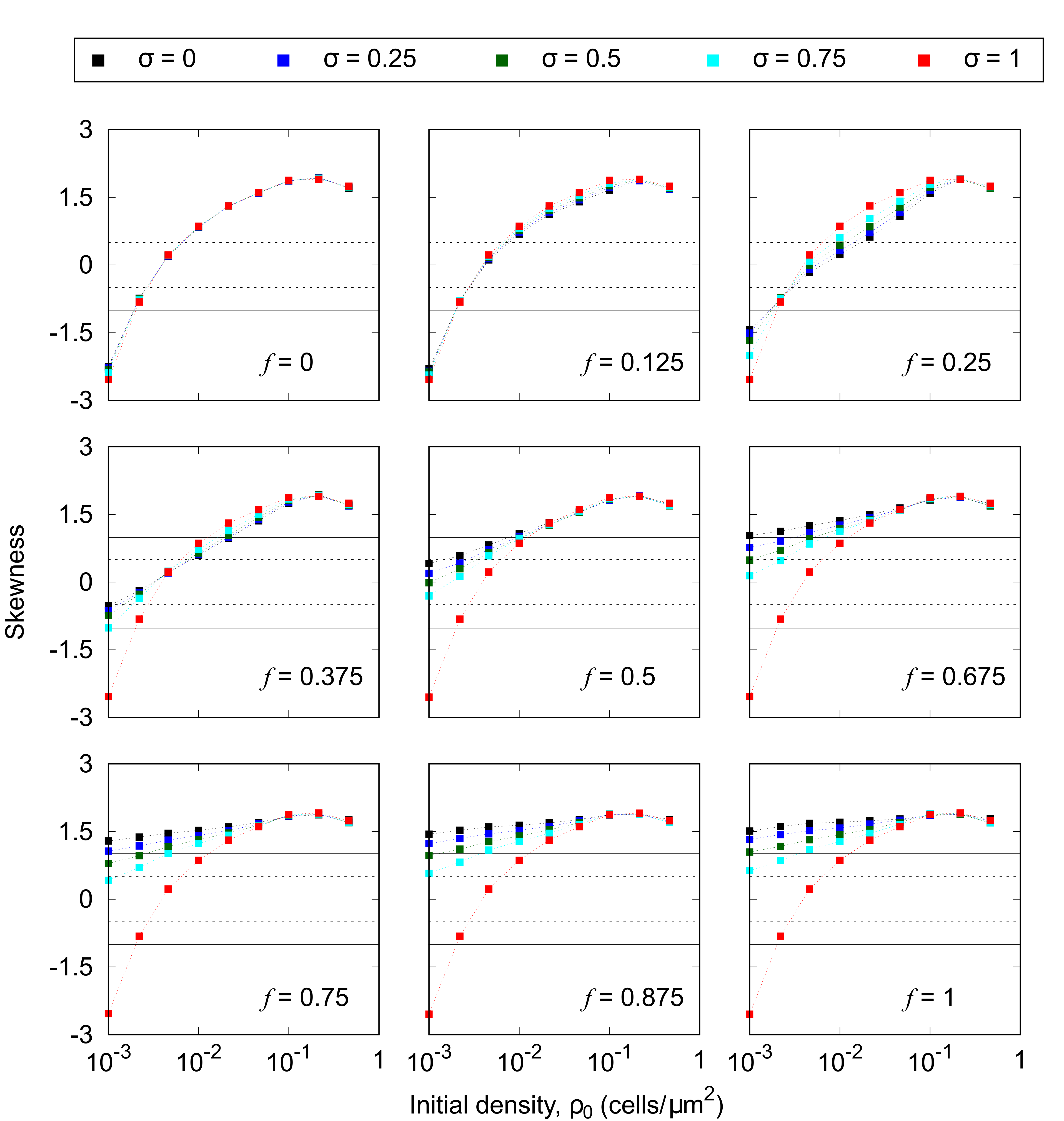

Supplement: S3 Fig — Skewness of the distribution of correlation lengths for different colonization strategies (σ, ρ0) and ecological conditions, given by the flow intensity f. Each curve represents a value of the adhesiveness σ, whose color code is maintained in all the panels. The skewness is obtained from 2x10^6^ independent realizations of the model. Horizontal dashed lines in each panel indicate the values +/- 0.5 and the full lines, +/- 1. Skewness in the interval [0.5, 1] in absolute value indicate that the data are moderately skewed, and if the skewness greater 1 in absolute value, then the distribution is highly skewed. (TIF) [file pcbi.1006094.s005.tif]

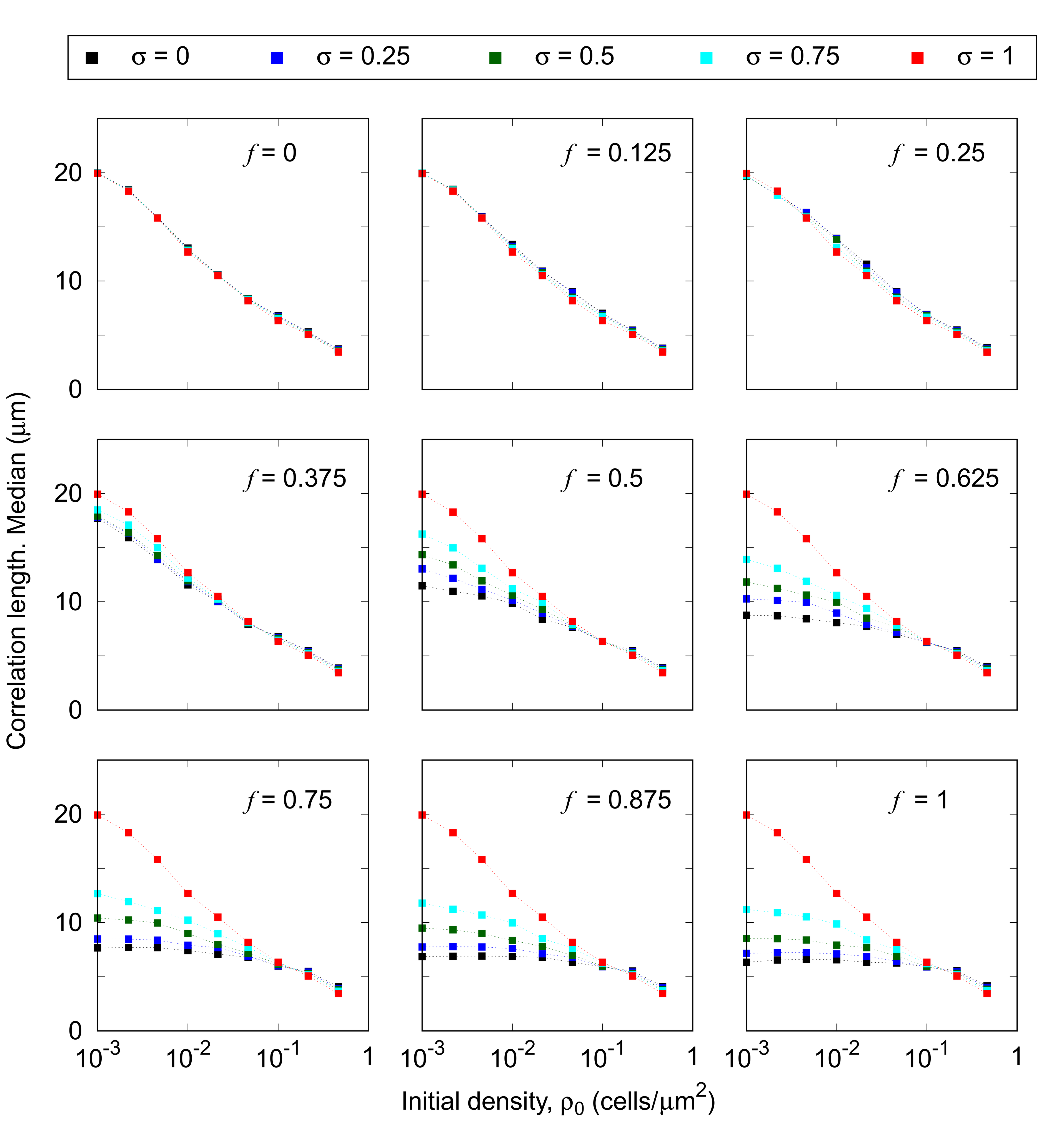

Supplement: S4 Fig — Median of the correlation length distribution for different colonization strategies (σ, ρ0) and ecological conditions given by the flow intensity f. Each curve represents a value of the adhesiveness σ. The color code is maintained in all the panels. The median is obtained from a set of 2x10^6^ independent model realizations. (TIF) [file pcbi.1006094.s006.tif]

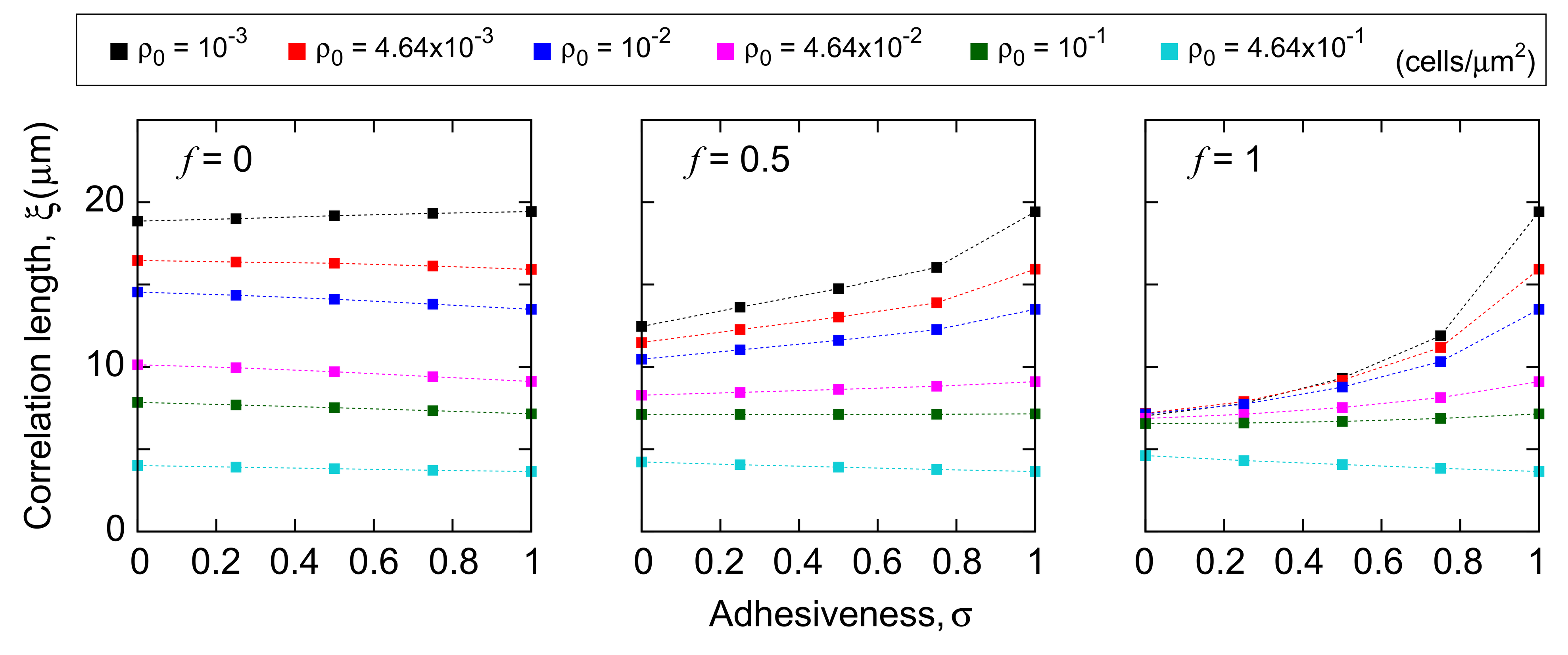

Supplement: S5 Fig — Mean correlation length, ξ, for different colonization strategies (σ, ρ0) in several ecological conditions given by the flow intensity f. Each curve represents a value of the initial density, ρ0. The color code is maintained in all the panels. Averages are taken over 2x10^6^ independent model realization. (TIF) [file pcbi.1006094.s007.tif]

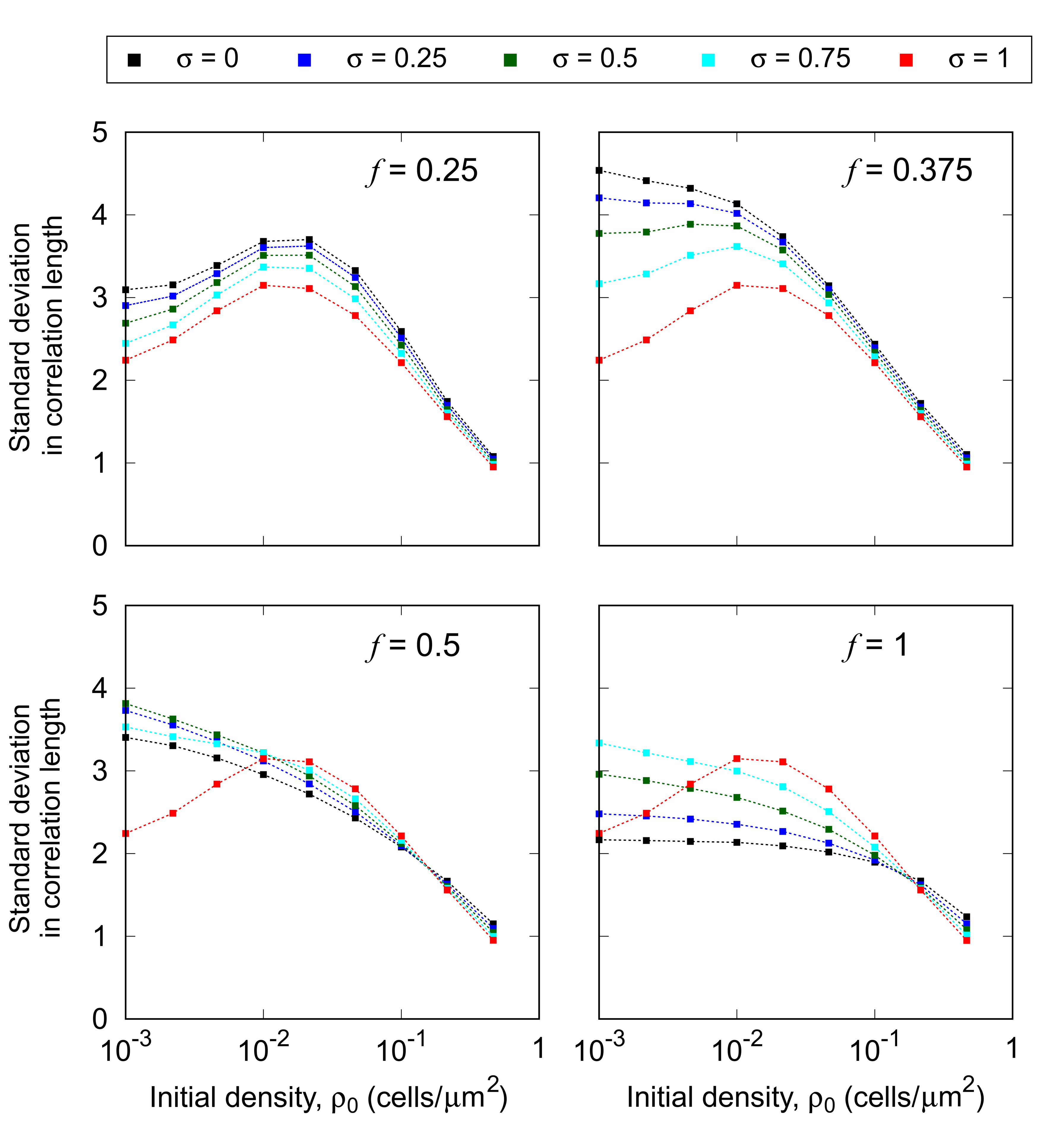

Supplement: S6 Fig — a) f = 0.25, b) f = 0.375, c) f = 0.5, d) f = 1. Each curve represents the standard deviation in ξ for a given adhesiveness, σ. Color code is maintained in all the panels. Averages are taken over 2x10^6^ independent model realizations. (TIF) [file pcbi.1006094.s008.tif]

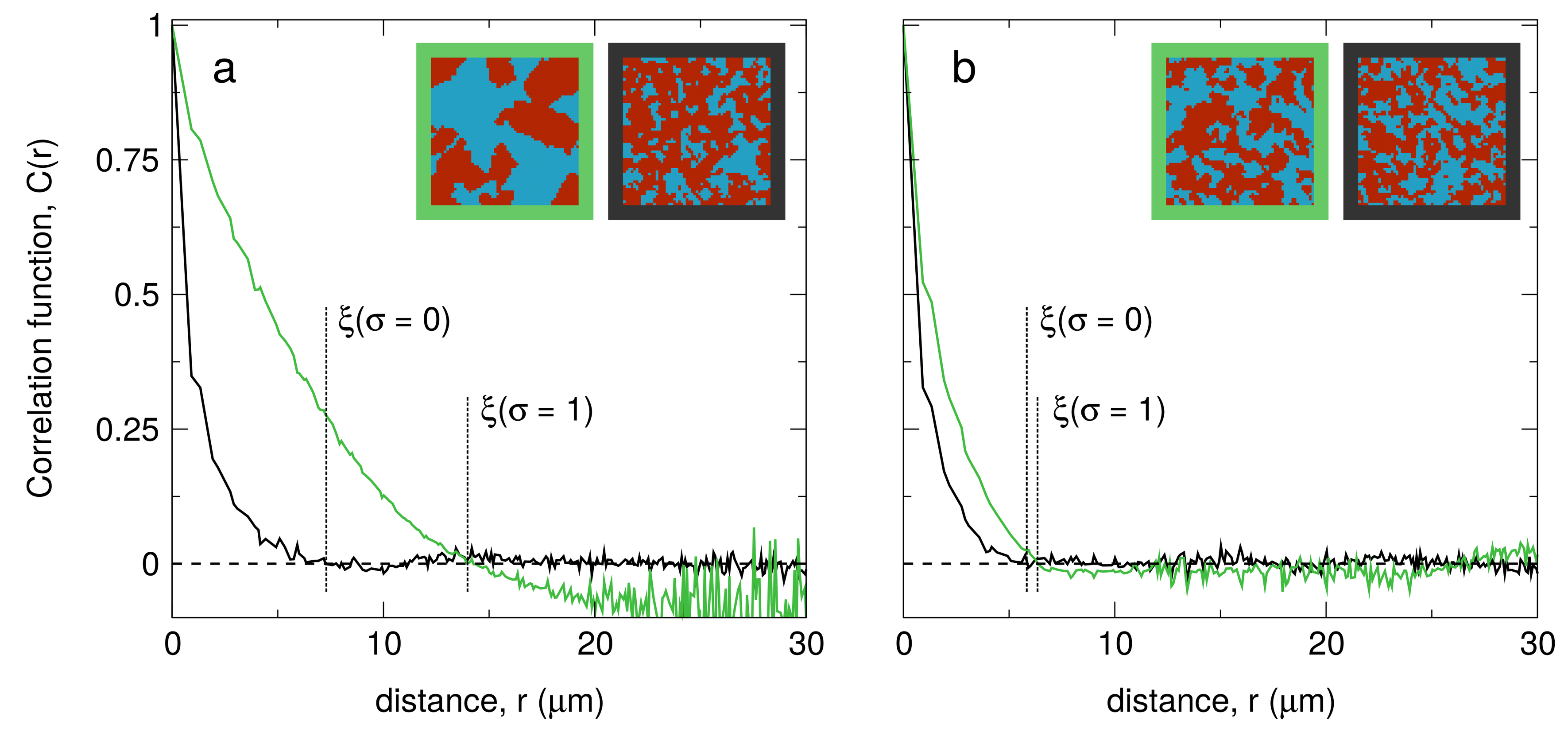

Supplement: S7 Fig — Correlation functions obtained for single realizations of the model at low (panel a; ρ0 = 10−3 cells/μm2) and high (panel b; ρ0 = 10−1 cells/μm2) initial density of cells. Correlation functions are obtained for the patterns shown in the snapshots. The color code indicates whether the pattern corresponds to σ = 1 (green) or σ = 0 (black) strains. The dashed lines point the value of the correlation length in each case, defined as the first zero of the correlation function. (TIF) [file pcbi.1006094.s009.tif]
